# Transcription Factor and Protein Regulatory Network of *PmACRE1* in *Pinus massoniana* Response to Pine Wilt Nematode Infection

**DOI:** 10.3390/plants13192672

**Published:** 2024-09-24

**Authors:** Wanfeng Xie, Xiaolin Lai, Yuxiao Wu, Zheyu Li, Jingwen Zhu, Yu Huang, Feiping Zhang

**Affiliations:** 1 Jinshan College, Fujian Agriculture and Forestry University, Fuzhou 350002, China; 000d080186@fafu.edu.cn (W.X.); 216725047@fafu.edu.cn (Z.L.); 2 Key Laboratory of Integrated Pest Management in Ecological Forests (Fujian Province University), Fujian Agriculture and Forestry University, Fuzhou 350002, China; 3210422029@fafu.edu.cn (X.L.); 5220422076@fafu.edu.cn (Y.W.); 52304022094@fafu.edu.cn (J.Z.); 3 Forestry College, Fujian Agriculture and Forestry University, Fuzhou 350002, China; 4 Fujian Academy of Forestry, Fuzhou 350000, China

**Keywords:** masson pine, pine wilt disease, *PmACRE1*, transcription factor, protein–protein interactions

## Abstract

Pine wilt disease, caused by *Bursaphelenchus xylophilus*, is a highly destructive and contagious forest affliction. Often termed the “cancer” of pine trees, it severely impacts the growth of Masson pine (*Pinus massoniana*). Previous studies have demonstrated that ectopic expression of the *PmACRE1* gene from *P. massoniana* in *Arabidopsis thaliana* notably enhances resistance to pine wilt nematode infection. To further elucidate the transcriptional regulation and protein interactions of the *PmACRE1* in *P. massoniana* in response to pine wilt nematode infection, we cloned a 1984 bp promoter fragment of the *PmACRE1* gene, a transient expression vector was constructed by fusing this promoter with the reporter *GFP* gene, which successfully activated the *GFP* expression. DNA pull-down assays identified PmMYB8 as a trans-acting factor regulating *PmACRE1* gene expression. Subsequently, we found that the PmACRE1 protein interacts with several proteins, including the ATP synthase CF1 α subunit, ATP synthase CF1 β subunit, extracellular calcium-sensing receptor (PmCAS), caffeoyl-CoA 3-O-methyltransferase (PmCCoAOMT), glutathione peroxidase, NAD+-dependent glyceraldehyde-3-phosphate dehydrogenase, phosphoglycerate kinase 1, cinnamyl alcohol dehydrogenase, auxin response factor 16, and dehydrin 1 protein. Bimolecular fluorescence complementation (BiFC) assays confirmed the interactions between PmACRE1 and PmCCoAOMT, as well as PmCAS proteins in vitro. These findings provide preliminary insights into the regulatory role of PmACRE1 in *P. massoniana*’s defense against pine wilt nematode infection.

## 1. Introduction

Pine wilt disease is a devastating forest epidemic in pine trees, which causes the yellowing and wilting of needles, stunted growth, premature needle drop, and the dieback of branches. The ultimate damage can lead to the death of the tree. Since its entry into Nanjing city in 1982, the disease has rapidly spread across multiple provinces, posing a serious threat to the ecological security of forests in China and causing significant economic losses [1]. Enhancing the disease resistance of Masson pine (*Pinus massoniana*) through effective technologies could mitigate the lethal effects of pine wood nematode (*Bursaphelenchus xylophilus*). Disease resistance in plants is closely related to the expression and regulation of resistance genes. Utilizing modern molecular techniques to uncover mechanisms of resistance in *P. massoniana* and identifying genes that regulate resistance capacity can expedite the development of resistant pine germplasm. This is crucial for the sustainable control of pine wilt disease [2,3,4].

Plant disease resistance is closely associated with the expression levels of disease-related genes. Numerous studies have demonstrated that different resistant varieties of *P. massoniana* show significant variations in the expression levels of genes involved in secondary metabolic pathways and antioxidant systems when infected by *B. xylophilus*. These genes potentially regulate *P. massoniana* resistance to the nematode [5,6,7,8]. Liu et al. found that enhancing the expression of terpene synthesis-related genes, such as *PmGPPS1*, *PmTPS4*, and *PmTPS21*, improves the plant defense against nematode infection [9,10], thereby confirming the efficacy of disease-resistance genes. Identifying key genes that regulate disease defense and employing molecular genetics to stably enhance their expression in *P. massoniana* could improve its resistance to pine wilt disease.

Our previous research identified differential gene expression in *P. massoniana* needles following *B. xylophilus* infection, with significant enrichment in pathways including phenylalanine metabolism, secondary metabolite biosynthesis, and plant hormone signal transduction [11,12]. Notably, the expression of the leucine-rich repeat (LRR) containing pathogen-specific recognition gene, Avr9/Cf-9 rapidly elicited (ACRE1), was markedly upregulated within a day post-infection. Additionally, genes associated with hypersensitive response pathways, such as plant–pathogen interactions, plant hormone signal transduction, and glutathione metabolism, exhibited altered expression patterns. With escalating nematode infection intensity, the expression of the *PmACRE1* gene in *P. massoniana* was suppressed, indicating a potential correlation between this gene and the tree’s resistance to nematode infection [12,13].

Upon pathogen infection, incompatible plant–pathogen interactions typically trigger a hypersensitive response (HR), leading to rapid local cell death mediated by the production of reactive oxygen species (ROS) and programmed cell death, which inhibits pathogen growth and enhances plant resistance [14]. Resistant pine varieties can activate the expression of ROS-related genes through HR, promoting the production of ROS to inhibit pine wood nematode infection [15]. *ACRE* genes, which are key players in mediating plant hypersensitive responses, have been extensively studied [16,17,18]. Durrant et al. first reported these genes using cDNA-AFLP technology to study tobacco defense responses mediated by Avr9 and cf-9, identifying 13 cDNA clones encoding *ACRE* genes induced by Avr9 in tobacco leaves. These ACRE proteins share homology with ethylene response elements, calcium-binding proteins, 13-lipoxygenase, and cyclo-H2 zinc finger proteins, making them readily inducible [19]. Ni et al. cloned a RING-H2 zinc finger protein gene (StRFP1) from potato, homologous to the *ACRE132* gene in tobacco. The overexpression of *StRFP1* in potato enhanced resistance to late blight, while suppression of *StRFP1* expression increased the susceptibility of transgenic potato plants to the disease [20]. De Vega et al. demonstrated that chitosan treatment induced the expression of two *ACRE* genes, *ACRE75* and *ACRE180*, in tomato early during pathogen infection, promoting callose deposition and jasmonate accumulation, thereby enhancing resistance to gray mold (*Botrytis cinerea*) [21]. These findings indicate that the expression intensity of ACRE genes mediates plant responses to pathogens and significantly influences their ability to resist pathogen invasion.

In conifers, the only reported *ACRE* gene is from Scots pine (*Pinus sylvestris*), where *PsACRE* regulates resistance to root rot [22]. The *PmACRE1* gene in *P. massoniana* encodes a 207 amino acid protein, differing from the ACRE protein in Scots pine by a single amino acid. It contains a leucine-rich repeat (LRR) motif, highlighting its conservation among pines. The LRR motif mediates protein–protein interactions, providing high-affinity domains for effective protein interaction sites, and is critical for the specific recognition of pathogen elicitors [22,23,24,25]. Previous studies have genetically transformed *PmACRE1* into *Arabidopsis*, demonstrating that transgenic *Arabidopsis* plants exhibit enhanced defense against pine wood nematode infection, reduced disease incidence, and increased levels of flavonoids, alkaloids, and triterpenoids in their leaves. Co-immunoprecipitation (Co-IP) studies revealed that the ACRE1 protein interacts with proteins involved in secondary metabolism and detoxification pathways in *Arabidopsis*, indicating that *PmACRE1* plays a role in defense against nematode infection and enhances plant resistance [26].

To further elucidate the regulatory mechanisms and protein interactions of PmACRE1 in *P. massoniana*, we cloned the flanking promoter region of *PmACRE1* from the *P. massoniana* genome and analyzed its cis-acting elements. A transient expression vector was constructed by fusing the promoter with the *GFP* gene to investigate promoter activity. DNA pull-down assays were performed to capture promoter-binding proteins, followed by mass spectrometry (MS) to identify and analyze associated transcription factors. Co-IP was employed to capture interacting proteins of PmACRE1, and bimolecular fluorescence complementation (BiFC) was used to validate these protein–protein interactions. These methodologies aim to enhance our understanding of the transcription factors regulating *PmACRE1* and the associated protein networks, providing theoretical insights into the transcriptional regulation of *PmACRE1* in response to pine wood nematode infection and paving the way for breeding disease-resistant *P. massoniana* varieties.

## 2. Materials and Methods

### 2.1. Plant Materials

The plant materials included 3-year-old *P. massoniana* grown in a greenhouse and *Nicotiana benthamiana* cultivated in a controlled climate chamber at 25 °C with a 16 h/8 h light/dark cycle, using peatmoss with vermiculite as a culture medium. The plant gene expression vectors p3301-*ΔActin*::GFP, p2300-35S::YFP^N^, and p2300-35S::YFP^C^ were provided by the Key Laboratory of Crop Ecology and Molecular Physiology, Fujian Agriculture and Forestry University [27].

### 2.2. Cloning and Analysis of the PmACRE1 Gene Promoter in P. massoniana

Genomic DNA was extracted from *P. massoniana* needles using the CTAB method [28]. The promoter region upstream of the *PmACRE1* gene’s initiation codon (ATG) was identified from the *P. massoniana* genome database (currently unreleased). Using genomic DNA as a template, a 1984 bp fragment upstream of the *PmACRE1* gene coding sequence (CDS) was amplified with primers *PmACRE1*-PRO-F (5′-GCTCATGGGTCTACTAACGGTTT-3′) and *PmACRE1*-PRO-R (5′-TTTTTTTGGGTTTTTTCTTCTGATC-3′) via TransTaq HiFi DNA Polymerase (TransGen Biotech Co., Ltd., Beijing, China). The purified amplified fragment was sequenced and bioinformatics analysis of the *PmACRE1* promoter sequence was performed using the PlantCARE online tool (http://bioinformatics.psb.ugent.be/webtools/plantcare/html/, accessed on 26 July 2024).

### 2.3. Detection of PmACRE1 Gene Promoter Activity

To assess the activity of the *PmACRE1* gene promoter, specific cloning primers incorporating sequences from the recombinant vector and Spe I and Sac I restriction sites were utilized: FGFP-*PmACRE1*-PRO-F (5′-atgattacgaattcGAGCTCGCTCATGGGTCTACTAACGG-3′; SpeI site underlined) and FGFP-*PmACRE1*-PRO-R (5′-tgtagtccatACTAGTTTTTTTTGGGTTTTTTCTTCTGATC-3′; SacI site underlined). A 1984 bp promoter sequence upstream of the *PmACRE1* gene was amplified using HiFi DNA Polymerase (TransGen Biotech Co., Ltd., Beijing, China). The PCR product was purified with the TIANquick Midi Purification Kit (TIANGEN Biotech (Beijing) Co., Ltd., Beijing, China). This purified promoter DNA was seamlessly cloned into a Spe I and Sac I linearized p3301-*ΔActin*::GFP vector using the pEASY-Basic Seamless Cloning and Assembly Kit (TransGen Biotech Co., Ltd., Beijing, China), resulting in the construction of the recombinant expression vector p3301-*PmACRE1*pro::GFP. This vector was transiently transformed into *N. benthamiana*, and the GFP expression driven by the *PmACRE1* promoter was analyzed using a Laser Scanning Confocal Microscope (Leica TCS SP8, Leica Microsystems, Wetzlar, Germany).

### 2.4. Isolation of Proteins Binding to the PmACRE1 Gene Promoter

Biotin was conjugated to the 5′ ends of the promoter amplification primers. The amplified *PmACRE1* gene promoter DNA was then purified. Natural soluble proteins were extracted from the *P. massoniana* needles, using the protein extraction method outlined by Fang et al. [28]. These proteins were incubated with the biotin-labeled *PmACRE1* gene promoter. The Dynabeads kilobaseBINDER Kit (Thermo Fisher Scientific Inc., Waltham, MA, USA) was employed to bind the biotin-labeled promoter–protein complexes to magnetic beads. Using a magnetic rack, the beads were isolated to retrieve the promoter-bound proteins. These proteins were subsequently separated by SDS-PAGE and compared with a negative control (incubated without the biotin-labeled promoter). Protein bands of interest were excised, digested with trypsin, and identified by Liquid Chromatography Mass Spectrometry/Mass Spectrometry (LC-MS/MS). The resulting mass spectrometry data were analyzed with Proteome Discoverer 2.5 software and searched against the UniProt protein database to identify the proteins and their functions, with a focus on transcription factors and DNA-binding proteins.

### 2.5. Subcellular Localization of Proteins Binding to the PmACRE1 Gene Promoter

Total RNA was extracted from *P. massoniana* needles using the RNAprep Pure Plant Plus Kit (Polysaccharides & Polyphenolics-rich, TIANGEN Biotech (Beijing) Co., Ltd., Beijing, China). The extracted RNA was reverse transcribed into cDNA with the EasyScript One-Step RT-PCR SuperMix kit (TransGen Biotech Co., Ltd., Beijing, China). The coding sequence (CDS) of the *PmMYB8* gene was used as a template for amplification, employing the following primers: FGFP-*PmMYB8*-F: 5′-gacaagacgcgtCCCGGGATGGGGCGCCACTCGTGC-3′ and FGFP-*PmMYB8*-R: 5′-aggtggaggtccCCCGGGAATTTGGTCCAGAACTG CAGC-3′ (Sma I site underlined). The amplified *PmMYB8* gene product was purified using the TIANquick Midi Purification Kit (TIANGEN Biotech (Beijing) Co., Ltd., Beijing, China). The purified *PmMYB8* gene fragment was then homologously recombined with the SmaI-linearized p3301-*ΔActin*::GFP vector using the pEASY-Basic Seamless Cloning and Assembly Kit (TransGen Biotech Co., Ltd., Beijing, China), resulting in the construction of the recombinant expression vector p3301-*PmACRE1*_PRO_::*PmMYB8*-GFP. This vector was transiently transformed into *N. benthamiana* to investigate the subcellular localization of the PmMYB8 protein.

### 2.6. Inoculation of Pine Wood Nematode to P. massoniana Seedlings and Proteins Extraction

*P*. *massoniana* seedlings were infected with pine wood nematode using the artificial bark inoculation method [29,30]. First, the base of the current year’s pruned branches was wiped with 70% ethanol. A sterile scalpel was used to make a slanted cut approximately 1 cm long, deep enough to reach the xylem. A sterile cotton ball was immediately placed in the wound. Using a sterile pipette tip, 0.2 mL of pine wood nematode suspension (500 nematodes) was inoculated onto the cotton ball. The wound was then gently wrapped and secured with sealing film. Sterile water was added every 2 h to maintain moisture. The seedlings were incubated in a greenhouse at 28 °C. Samples of *P. massoniana* needles were collected on the 3rd and 5th days post-infection for the extraction of natural soluble proteins [27]. The extracted protein solution was stored at −80 °C.

### 2.7. Co-IP for Isolation of PmACRE1 Interacting Proteins

Interacting proteins of PmACRE1 were isolated using the BeaverBeads Protein A/G Immunoprecipitation Kit (Beaver Biosciences Inc., Suzhou, China). BeaverBeads Protein A/G magnetic beads were first incubated with a specific antibody against PmACRE1 at 26 °C for 60 min to allow antibody binding. The antibody–bead complex was then incubated overnight at 4 °C with natural soluble proteins extracted from *P. massoniana* needles collected 3- and 5-days post-pine wood nematode infection. This enabled the isolation of PmACRE1 along with its interacting proteins. The eluted protein complexes were subjected to SDS-PAGE and stained with Coomassie Brilliant Blue. Protein bands were excised, digested, and analyzed by HPLC-MS/MS. The data were then analyzed using Proteome Discoverer software and searched against the UniProt database to identify protein information and functionality.

### 2.8. KEGG Enrichment Analysis for PmACRE1-Interacting Proteins

The FASTA file containing the *P. massoniana* gene database was obtained from the NCBI database. Gene IDs were extracted and converted using the TBtools-II software. The background set was annotated through the KEGG online website. Subsequently, R language (R-4.3.2), R studio (2021.09.0+351), and necessary compilers were downloaded and installed. The clusterProfiler package 4.0 was loaded into the environment, and the appropriate code was executed to conduct KEGG enrichment analysis. This process culminated in the generation of visualizations to elucidate the functional pathways associated with the PmACRE1-interacting proteins.

### 2.9. BiFC Assay for Validating Interactions among PmCCoAOMT, PmCAS, and PmACRE1

Specific amplification primers were designed for PmCCoAOMT, PmCAS, and PmACRE1, incorporating the Spe I site (underlined). The sequences for PmCCoAOMT-2YNC-F and -R were 5′-atggcgcgccACTAGTATGGCAAGCACAGATGTTGCTG-3′ and 5′-cacctcctccACTAGTATAGACACGCCT GCAAAGGGT-3′, respectively. Similarly, primers for PmCAS-2YNC-F and -R were 5′-atggcgcgccACTAGTATGGCTAGCAAGGCAGTTGGC-3′ and 5′-cacctcctccACTAGTATCATCCAAACCACTAGCAAGAA-3′, and for PmACRE1-2YNC-F and -R were 5′-atggcgcgccACTAGTATGGAGGTCCATTCTACTGT AAAT-3′ and 5′-cacctcctccACTAGTGGCTGTACCCCTTTCAAGCATCT-3′. Using cDNA from *P. massoniana* needles as a template, the genes *PmCCoAOMT*, *PmCAS*, and *PmACRE1* were amplified, purified, and sequenced. These genes were then individually cloned into the YFP-tagged vectors p2300-2YN and p2300-2YC, which had been linearized with the restriction endonuclease SpeI. The resulting recombinant expression vectors were utilized in a tobacco transient expression system mediated by Agrobacterium. This system allowed for the co-expression of *PmACRE1* with either *PmCCoAOMT* or *PmCAS*. The interactions between PmACRE1 and PmCCoAOMT, as well as between PmACRE1 and PmCAS, were visualized and confirmed using a Laser Scanning Confocal Microscope (Leica TCS SP8, Leica Microsystems, Wetzlar, Germany).

## 3. Results

### 3.1. PmACRE1 Gene Promoter and Its Cis-Elements

In this study, a 1984 bp DNA fragment upstream of the *PmACRE1* gene open reading frame was isolated. Sequencing and alignment confirmed its location upstream of the *PmACRE1* gene initiation codon. Analysis using the PlantCARE online tool identified 38 cis-acting elements within the *PmACRE1* promoter sequence (see Supplementary Figure S1). The promoter contains binding sites for transcription factors such as MYB, MYC, and Myb (MBS), along with elements involved in specific transcriptional regulatory processes (e.g., AE-box, TCA-element, TCCC-motif, and TGA-element), gene transcription activation (CCAAT-box), stress response (STRE), ethylene responsiveness (ERE), gibberellin responsiveness (P-box), abscisic acid responsiveness (ABRE, ABRE3a, ABRE4), low-temperature responsiveness (LTR), light responsiveness (G-Box), anaerobic responsiveness (ARE), and cis-acting element for WRKY (W box) related to plant immune and pathogen infection responses, etc. Additionally, elements related to anthocyanin synthesis regulation (chs-CMA1a) and circadian rhythm regulation (circadian) were identified (see Table 1). Several response elements of unknown function, such as the AAGAA-motif, Unnamed_1, Unnamed_2, and Unnamed_4, were also identified (see Table 1), suggesting that this promoter is regulated by a variety of factors, including light, temperature, plant hormones, pathogens, and stress conditions.

### 3.2. Activity of the PmACRE1 Gene Promoter

**Figure 1:**
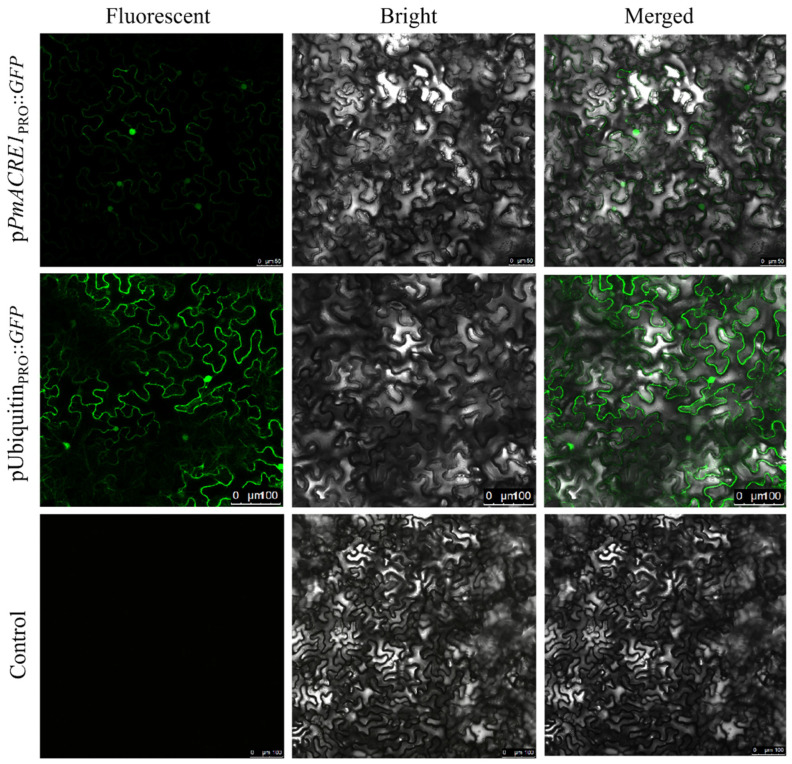
Transient expression of *PmACRE1* gene promoter-driven GFP expression in *N. benthamiana* leaves.

Using an Agrobacterium-mediated transient transformation system in *Nicotiana benthamiana* leaves, the *PmACRE1*_PRO_::GFP recombinant vector was transiently expressed. Tobacco leaves inoculated with an empty GFP vector served as controls. As shown in Figure 1, the *PmACRE1*_PRO_::GFP recombinant protein was expressed in both the cell membrane and the nucleus of the tobacco leaves. This observation confirms that the *PmACRE1* promoter is active and capable of driving the expression of the reporter gene *GFP*.

### 3.3. DNA-Binding Proteins of the PmACRE1 Gene Promoter

Identification of DNA-binding proteins associated with the *PmACRE1* gene promoter was achieved using DNA pull-down assays from natural soluble proteins extracted from *P. massoniana* needles (see Supplementary Figure S2). Mass spectrometry analysis revealed a total of 284 binding proteins (see Supplementary Table S1). Key proteins interacting with the promoter DNA segment included the PmMYB8 transcription factor, ribosomal protein, photosystem II protein K (plastid), propene double bond reductase, glutathione S-transferase, type I chlorophyll a/b binding protein, and ATP synthetase CF1β subunit (chloroplast), among others (see Table 2). Notably, PmMYB8 is a recognized transcription factor that potentially regulates *PmACRE1* gene expression.

### 3.4. Subcellular Localization of the PmMYB8 Transcription Factor

**Figure 2:**
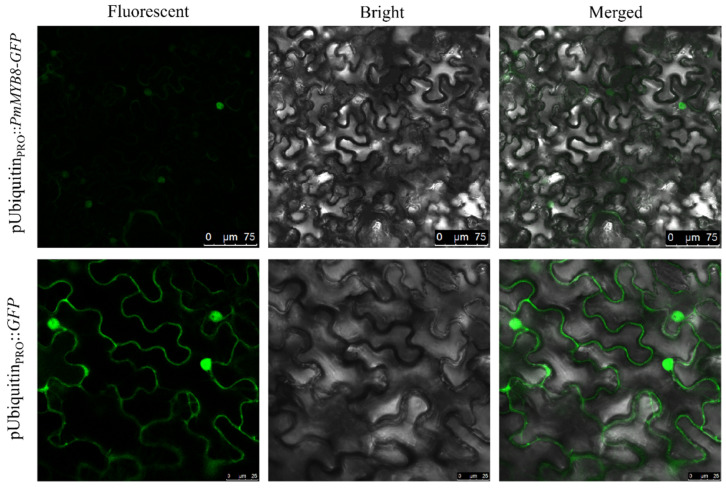
Subcellular localization of PmMYB8 transcription factor in *N. benthamiana* leaves.

The *PmMYB8* gene was cloned to construct a recombinant expression vector fused with *GFP* for transient transformation in *N. benthamiana* to investigate the subcellular localization of PmMYB8. The results showed green fluorescence localized in the nucleus, whereas the control empty GFP vector exhibited expression both on the cell membrane and in the nucleus (see Figure 2). These findings indicate that the PmMYB8-GFP fusion protein is specifically localized in the nucleus, consistent with the characteristics of transcription factors.

### 3.5. Proteins Interacting with PmACRE1

**Figure 3:**
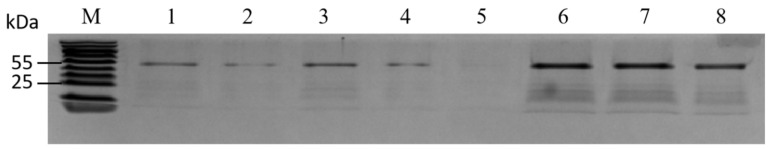
Detection of Proteins Interacting with PmACRE1. M, Protein ladder; Lanes 1–8, Proteins binding on the *PmACRE1* gene promoter.

The pine wood nematode can colonize pine needles and continue to reproduce, and it can also spread to pine twigs through these needles [31]. To further investigate the regulatory network of PmACRE1 protein in Pine wood Nematode-infected *P. massoniana*, specific antibodies against PmACRE1 were prepared. Interacting proteins of PmACRE1 were isolated from the natural soluble proteins of *P. massoniana* needles (see Supplementary Figure S3) using Protein A+G Magnetic Beads-based Co-IP. The PmACRE1 protein and its potential interacting proteins were separated by SDS-PAGE (see Figure 3). Coomassie Brilliant Blue staining revealed that in addition to the PmACRE1 protein, several potential interacting proteins were detected, indicating a complex regulatory network involving PmACRE1 in response to pinewood nematode infection.

To elucidate the types and functions of PmACRE1-interacting proteins, mass spectrometry was performed, identifying 42 proteins detailed in Table 3. These include PmACRE1, ATP synthase CF1 alpha and beta subunits, extracellular calcium-sensing receptor (PmCAS), caffeoyl-CoA 3-O-methyltransferase (PmCCoAOMT), glutathione peroxidase (GSH-Px), NAD^+^-dependent glyceraldehyde-3-phosphate dehydrogenase, auxin response factor 16 (ARF16), phosphoglycerate kinase 1, cinnamyl alcohol dehydrogenase (PmCAD), and dehydrin 1 protein, among others. These findings suggest that PmACRE1 may play a role in defense against pinewood nematode disease by interacting with these proteins. Notably, PmCCoAOMT, PmCAD, PmCAS, and NAD+-dependent glyceraldehyde-3-phosphate dehydrogenase are involved in secondary metabolic pathways in plants, indicating that PmACRE1 may regulate these pathways through its interactions.

### 3.6. KEGG Enrichment Analysis of PmACRE1 Interacting Proteins in P. massoniana

**Figure 4:**
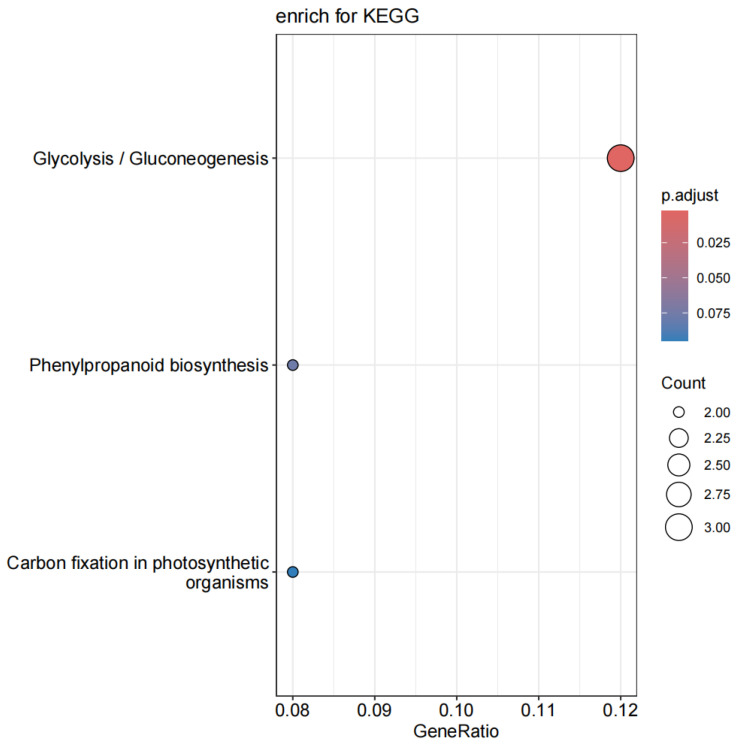
KEGG enrichment analysis of PmACRE1-interacting proteins in *P. massoniana.*

Pathway enrichment analysis of PmACRE1-interacting proteins was conducted using the KEGG database. The results indicated that these proteins are enriched in pathways such as Glycolysis/Gluconeogenesis, Phenylpropanoid biosynthesis, and Carbon fixation in photosynthetic organisms (see Figure 4).

### 3.7. BiFC Validation of Interactions between PmACRE1 and PmCCoAOMT, PmCAS

**Figure 5:**
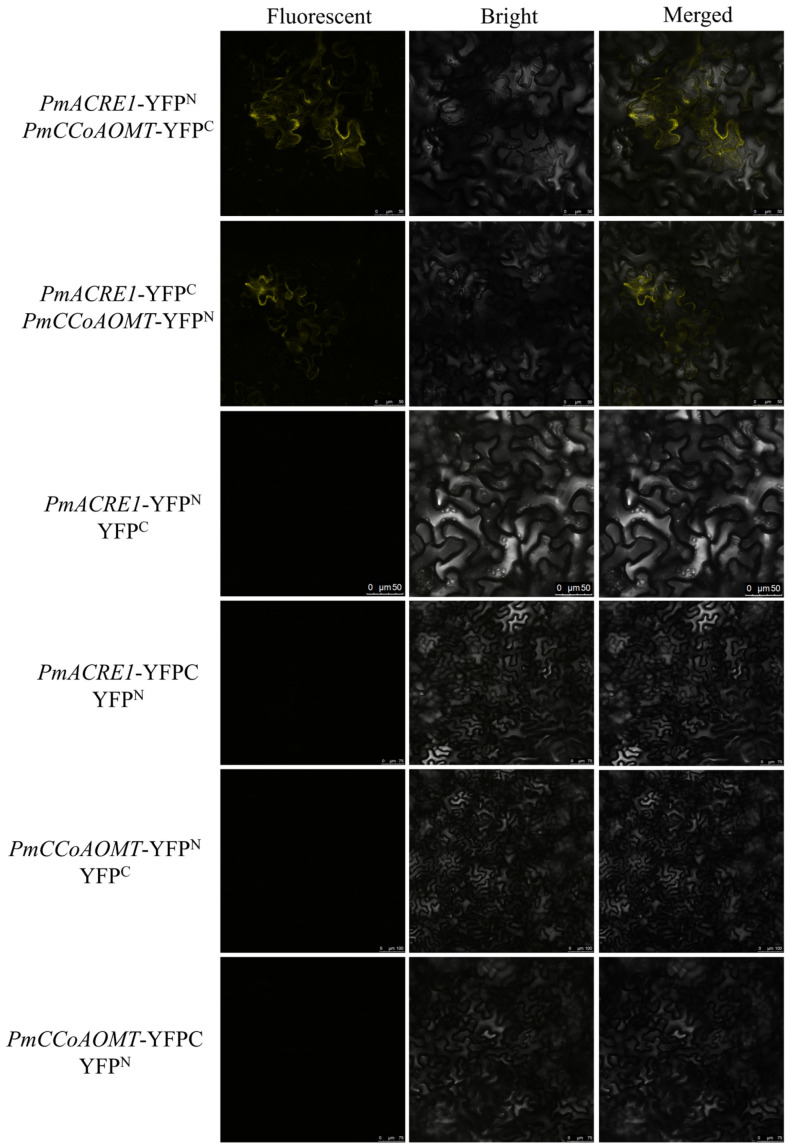
BiFC Validation of Protein Interaction between PmACRE1 and PmCCoAOMT.

**Figure 6:**
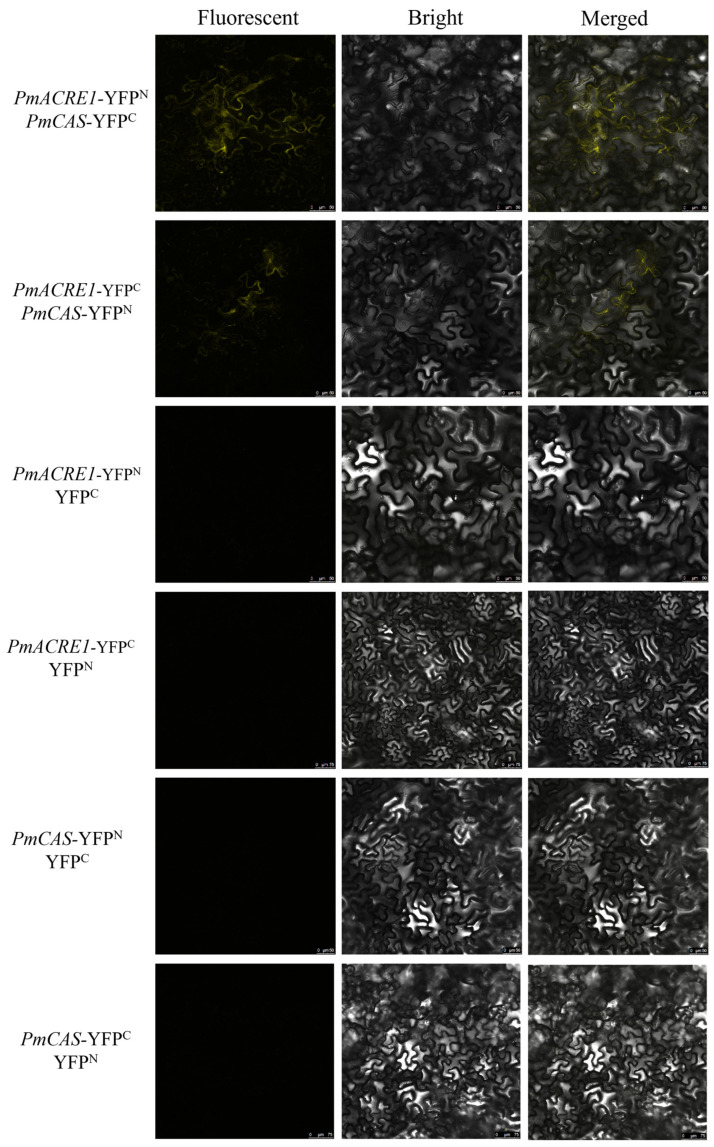
BiFC Validation of Protein Interaction between PmACRE1 and PmCAS.

The interactions between PmACRE1 and proteins involved in plant disease resistance-related secondary metabolic pathways, specifically PmCCoAOMT and PmCAS, were validated using Bimolecular Fluorescence Complementation (BiFC) technology. The coding sequence (CDS) of the CCoAOMT gene was cloned and fused with the N-terminal and C-terminal domains of Yellow Fluorescent Protein (YFP), resulting in the construction of PmCCoAOMT-YFP^N^ and PmCCoAOMT-YFP^C^ recombinant expression vectors. Similarly, PmACRE1-YFP^N^ and PmACRE1-YFP^C^ vectors were also constructed. These vectors were transiently expressed in *N. benthamiana* leaves, along with a negative control group consisting of YFP^N^ and YFP^C^ without the target genes. Confocal microscopy revealed yellow fluorescent signals in *N. benthamiana* leaves co-expressing PmACRE1-YFP^N^ with PmCCoAOMT-YFP^C^, PmACRE1-YFP^C^ with PmCCoAOMT-YFP^N^, as well as PmACRE1-YFP^N^ with PmCAS-YFP^C^, and PmACRE1-YFP^C^ with PmCAS-YFP^N^ (see Figure 5 and Figure 6). No fluorescent signals were observed in the negative control group. These results confirm the interactions between PmACRE1 and PmCCoAOMT, as well as PmACRE1 and PmCAS.

## 4. Discussion

Pine wood nematode infection triggers notable gene expression changes in *P. massoniana*, including genes involved in flavonoid biosynthesis, plant hormone signal transformation, amino sugar and nucleoside sugar metabolism, and MAPK signaling pathways [32]. Similarly, in Japanese red pine, defense response genes, secondary metabolism genes, transcriptional regulation genes, pathogenesis-related proteins, pinene synthases, and metallothioneins were upregulated following nematode inoculation [33]. Early-stage resistance responses, such as oxidative stress, lignin synthesis, ethylene production, and post-transcriptional mRNA regulation, were activated in the pine trees post-infection [34]. Thus, identifying and validating key genes for their role in disease resistance is crucial. A QTL analysis using a genetic linkage map and phenotypic data from a PWN inoculation test identified the *PWD1* locus as a major resistance QTL on Pinus consensus LG03, conferring pine wood nematode resistance dominantly [35]. Transcriptomic analysis indicates that ROS and phenylpropanoid metabolism, particularly lignin synthesis, are crucial for pine resistance to PWN. This is evidenced by the upregulation of genes in phenylpropanoid and lignin synthesis pathways, with cinnamoyl-CoA reductase genes being upregulated in PWN-resistant *P*. *thunbergii* and downregulated in PWN-susceptible *P*. *thunbergii*. Additionally, lignin content is consistently higher in resistant *P*. *thunbergii* compared to susceptible ones [36].

Our previous studies showed that the *PmACRE1* (Avr9/Cf-9 rapidly elicited) gene is downregulated during pathogen infection in *P. massoniana*, indicating its possible role in disease resistance [12]. Transgenic *Arabidopsis* plants expressing *PmACRE1* exhibited enhanced defense against pine wood nematodes. The PmACRE1 protein interacts with proteins involved in secondary metabolism, detoxification, stress response, and primary metabolism, regulating the synthesis of secondary metabolites, promoting flavonoid and phenolic compound production, and increasing APX enzyme activity, thereby enhancing plant resistance [26].

Gene expression regulation is a highly complex process. Transcription factors (TFs) bind to cis-regulatory elements in promoter regions, acting as trans-acting factors to regulate downstream stress-responsive genes. This regulation enables TFs to induce molecular, physiological, and biochemical adjustments in plants, facilitating defense responses to various stresses [37]. Kuang et al. (2020) used WGCNA and cis-motif enrichment to construct a TF regulatory network for ethylene-mediated banana ripening, identifying 25 TFs involved in regulating ripening-related genes with mutual interactions [38]. Zhu et al. (2023) employed DAP-seq to identify the binding sequence of an AP2/ERF TF and its target gene ZmEREB57 in maize [39]. Xie et al. (2023) combined DNA pull-down with LC/MS to identify 31 TFs interacting with CCRM-1 in wheat, showing that TaB3-2A1 binding to CCRM-1 regulates starch content, nitrogen homeostasis, and traits like heading date, plant height, and grain weight [40]. In this study, we cloned a 1984 bp fragment of the *PmACRE1* gene promoter from *P. massoniana* and identified the transcription factor PmMYB8, which regulates *PmACRE1* expression.

MYB transcription factors are pivotal regulatory elements in plants, influencing growth, development, metabolism, and environmental responses. Based on conserved amino acids in their DNA-binding domains, MYB transcription factors are categorized into 1R-MYB/MYB-related, R2R3-MYB, 3R-MYB, and 4R-MYB [41]. The R2R3-MYB subfamily, the largest within the MYB family, regulates secondary metabolism, hormone signaling pathways, and defense mechanisms. Craven et al. (2013) identified AC elements in the promoters of genes encoding phenylalanine aminotransferase (PAT), phenylalanine ammonia-lyase (PAL), and glutamine synthetase (GS1b), all involved in R2R3-MYB-mediated transcriptional activation. The R2R3-MYB transcription factor MYB8 regulates the expression of PAT, PAL, and GS1b, thus participating in phenylalanine metabolism [42]. Pandey et al. (2012) reported that ectopic expression of *AtMYB12* in tobacco increased the expression of key enzyme genes in the phenylpropanoid pathway, enhancing flavonoid biosynthesis [43]. Yu et al. (2023) demonstrated that transgenic foxtail millet overexpressing *SiMYB16* exhibited higher flavonoid and lignin contents and enhanced fatty acid synthase activity under salt stress, suggesting *SiMYB16* positively regulates salt tolerance by modulating lignin and suberin biosynthesis [44]. Liu et al. (2024) identified *PbrMYB4* as a key gene related to stone cell content in pear fruit through eQTL and gene co-expression network analysis. Genetic transformation confirmed that PbrMYB4 promotes lignin synthesis in pear fruit, callus, and *Arabidopsis* stems by directly activating 4CL1 expression through binding to the AC-I cis-element [45].

A cell-type-specific protein–protein interaction mediates cellular responses. In *Arabidopsis*, the interaction between the SM protein SEC1A and the Exocyst subunit SEC6 involves mutual regulation during pollen tube growth [46]. The pepper JAZ protein CaJAZ1-03 negatively regulates drought and ABA signaling, with its stability controlled by the RING-type E3 ubiquitin ligase CaASRF1 [47]. This study identified that the PmACRE1 protein interacts with various proteins enriched in metabolic pathways, such as glycolysis/gluconeogenesis, phenylpropanoid biosynthesis, and carbon fixation. Key interacting proteins include caffeoyl-CoA O-methyltransferase (CCoAOMT), extracellular calcium sensing receptor (CAS), glutathione peroxidase (GSH-Px), and auxin response factor (ARF16). CCoAOMT plays a pivotal role in lignin biosynthesis, a crucial component of plant cell walls that provides structural stability and support [48]. Lignin is essential for secondary metabolic regulation, influencing plant growth, development, and responses to environmental stress [49]. The CCoAOMT role in lignin synthesis underscores its importance in these processes.

Wagner et al. (2011) isolated a cDNA clone encoding the lignin-related enzyme CCoAOMT from a radiata pine differentiating xylem cDNA library [50]. Zhang et al. (2015) cloned the CCoAOMT gene from tea and analyzed its expression in different tea cultivars and leaf maturation stages, confirming its role in catalyzing EGCG methylation [51]. In *Arabidopsis*, CCoAOMT T-DNA insertion mutants showed reduced lignin content in stems, with lower G-lignin content and a higher S-lignin/G-lignin ratio compared to wild-type plants [52]. Similarly, silencing CCoAOMT in transgenic flax reduced lignin content and altered S-lignin/G-lignin ratios, accompanied by changes in xylem tissue structure [53].

Our study confirmed the interaction between PmACRE1 and PmCCoAOMT, implying that PmACRE1 may influence lignin synthesis via the phenylpropanoid biosynthesis pathway. Additionally, we identified the interaction between PmACRE1 and PmCAS, a protein located on thylakoid membranes. CAS senses extracellular calcium ions and activates signaling pathways critical for stress responses. Jia et al. found that the expression of three CAS homologs and chloroplast genes in wheat is suppressed following fungal infection or fusaric acid application, suggesting that these genes may reduce fusaric acid sensitivity in wheat [54]. Nomura et al. showed that the CAS signaling pathway in chloroplasts affects PTI and ETI in *Arabidopsis* treated with flg22. RNA-Seq results indicated that CAS regulates the expression of PAMP-induced defense genes and suppresses chloroplast gene expression, possibly via retrograde signaling mediated by singlet oxygen (^1^O_2_), highlighting chloroplast-mediated transcriptional reprogramming in plant immune responses [55,56]. Therefore, ACRE1 may interact with CAS to utilize calcium signaling, triggering defense responses in *P. massoniana*.

Additionally, glutathione peroxidase (GSH-Px) and auxin response factors (ARFs) also participate in the plant’s response to stress. Under stress conditions, GSH-Px enhances plant survival and adaptability by eliminating harmful peroxides and participating in signal transduction processes [57,58]. Auxins such as indole-3-acetic acid (IAA), which are produced in new buds, shoots, and canopy foliage, and can be transported polarialy down the stem, may be sufficient to trigger cambium cell differentiation [59,60]. ARFs are transcription factors that interact with the auxin/indole-3-acetic acid repressors (Aux/IAAs). Upon auxin binding, it is released from these repressors, as auxin induces the degradation of Aux/IAAs, thereby activating the transcription of ARF-targeted genes [61,62]. Additionally, IAA content was highest in the needle leaves surrounding the apical buds [63]. Multiple studies have demonstrated that ARF proteins are essential in enhancing plant stress tolerance [64,65,66].

In summary, this study has identified the transcriptional regulators of the disease-resistance gene *PmACRE1* in *P. massoniana* and elucidated the protein regulatory network involving PmACRE1. The PmACRE1 protein likely interacts with PmCCoAOMT, PmCAS, GSH-Px, ARF16, and other proteins, influencing metabolic pathways such as glycolysis/gluconeogenesis, phenylpropanoid biosynthesis, and carbon fixation. Through these interactions, ACRE1 may regulate *P. massoniana* resistance to pine wood nematode infestation. This comprehensive understanding of the molecular mechanisms by which PmACRE1 enhances resistance provides a theoretical foundation for leveraging these genes to improve pine tree disease resistance.

## Figures and Tables

**Table 1:** Regulatory cis-elements identified in the *PmACRE1* gene promoter sequence.

Cis-Regulatory Element	Core Sequence	Totals	Function
AAGAA-motif	gGTAAAGAAA/GAAAGAA	3	Unknown
ABRE	CACGTG/CACGTG	3	cis-acting element involved in the abscisic acid responsiveness
ABRE3a	TACGTG	1	cis-acting element involved in abiotic stress and signaling pathway
ABRE4	CACGTA	1	cis-acting element involved in abiotic stress and signaling pathway
AE-box	AGAAACTT	1	part of a module for light response
ARE	AAACCA	5	cis-acting regulatory element essential for the anaerobic induction
AT~TATA-box	TATATAAA	3	involved in the formation of a transcription initiation complex
CAAT-box	CAAAT/CAAT	39	common cis-acting element in promoter and enhancer regions
CCAAT-box	CAACGG	1	MYBHv1 binding site
ERE	ATTTTAAA	1	cis-acting element involved in the response to ethylene
G-Box	CACGTG	2	cis-acting regulatory element involved in light responsiveness
G-box	TAACACGTAG/GCCACGTGGA/TACGTG/CACGTG	4	cis-acting regulatory element involved in light responsiveness
GT1-motif	GGTTAA	1	light responsive element
LTR	CCGAAA	1	cis-acting element involved in low-temperature responsiveness
MBS	CAACTG	2	MYB binding site involved in drought-inducibility
MRE	AACCTAA	1	MYB binding site involved in light responsiveness
MYB	CAACAG/CAACCA/TAACCA	6	MYB binding site
MYB recognition site	CCGTTG	1	MYB binding site
MYB-like sequence	TAACCA	3	MYB binding site
MYC	CAATTG/CATTTG	2	basic helix-loop-helix (bHLH) binding motifs
Myb	CAACTG	2	MYB binding site
Myb-binding site	CAACAG	2	MYB binding site
O2-site	GATGATGTGG	1	cis-acting regulatory element involved in zein metabolism regulation
P-box	CCTTTTG	1	gibberellin-responsive element
STRE	AGGGG	2	involved in peroxisome biogenesis, function, and regulation
TATA	TATAAAAT	2	involved in the formation of a transcription initiation complex
TATA-box	TATATAA	45	core promoter element around −30 of transcription start
TCA-element	CCATCTTTTT	2	cis-acting element involved in salicylic acid responsiveness
TCCC-motif	TCTCCCT	1	part of a light responsive element
Unnamed_1	CGTGG	2	Unknown
Unnamed_2	AACCTAACCT	1	Unknown
Unnamed_4	CTCC	6	Unknown
W box	TTGACC	1	a core sequence acts as a binding site for WRKY TFs
WUN-motif	AAATTACT/TTATTACAT	2	wound-responsive element
chs-CMA1a	TTACTTAA	1	part of a light responsive element
circadian	CAAAGATATC	1	cis-acting regulatory element involved in circadian control

**Table 2:** Identification of binding proteins of the *PmACRE1* gene promoter in *Pinus massoniana*.

Accession	Description	Sum PEP Score	Peptides	Unique Peptides
AIZ74346.1	phosphoglycerate kinase 1 [*Pinus massoniana*]	109.786	26	26
AHL24663.1	ribulose-1,5-bisphosphate carboxylase/oxygenase activase large isoform [*Pinus massoniana*]	116.557	23	23
ULQ63856.1	ATP synthase CF1 beta subunit (chloroplast) [*Cuscuta japonica*]	47.272	12	2
AIZ74323.1	actin related protein 1 [*Pinus massoniana*]	28.62	11	11
QEP51812.1	elongation factor [*Pinus massoniana*]	35.434	10	1
AIZ74328.1	translation elongation factor 1-alpha [*Pinus massoniana*]	33.002	10	1
AFA51418.1	extracellular calcium sensing receptor [*Pinus massoniana*]	28.98	10	10
AGC13142.1	DHAR class glutathione S-transferase [*Pinus tabuliformis*]	23.517	9	9
ADV40957.1	caffeoyl-CoAO-methyltransferase [*Pinus radiata*]	17.378	7	7
AIZ74331.1	alpha-tubulin [*Pinus massoniana*]	14.751	7	6
AGT98543.1	glutathione peroxidase 2 [*Pinus tabuliformis*]	13.11	6	4
AIZ74330.1	cyclophilin [*Pinus massoniana*]	14.913	5	5
QSD59059.1	heat shock 90 kDa protein [*Pinus sylvestris*]	10.745	5	5
CAA41404.1	Type 1 chlorophyll a /b-binding protein [*Pinus sylvestris*]	7.835	2	2
ACJ70336.1	putative ribosomal protein S10, partial [*Pinus sylvestris*]	4.348	2	1
AGC13149.1	phi class glutathione S-transferase [*Pinus tabuliformis*]	3.945	2	2
AHA90706.1	aquaporin [*Pinus massoniana*]	3.003	2	2
CBM40481.1	MYB8 transcription factor [*Pinus pinaster*]	0.752	1	1
ACL14200.1	putative ribosomal protein L34, partial [*Pinus sylvestris*]	0.749	1	1
AXQ01589.1	photosystem II protein K (plastid) [*Pinus pinea*]	0.714	1	1
YP_008082259.1	ribosomal protein S12 (chloroplast) [*Pinus massoniana*]	0.674	1	1

**Table 3:** Identification of PmACRE1 interacting proteins in *P. massoniana.*

Accession	Description	Sum PEP Score	Peptides	Unique Peptides
AHL24663.1	ribulose-1,5-bisphosphate carboxylase/oxygenase activase large isoform [*Pinus massoniana*]	68.19	23	23
WCL24039.1	ribulose-1,5-bisphosphate carboxylase/oxygenase large subunit (chloroplast) [*Pinus massoniana*]	30.174	13	13
QEP51812.1	elongation factor [*Pinus massoniana*]	18.672	8	2
ASU09148.1	disease resistance protein [*Pinus massoniana*]	21.065	7	7
AIZ74328.1	translation elongation factor 1-alpha [*Pinus massoniana*]	16.688	7	1
WCL23812.1	cytochrome f (chloroplast) [*Pinus massoniana*]	14.926	7	7
AFA51418.1	extracellular calcium sensing receptor [*Pinus massoniana*]	13.155	17	6
AIZ74323.1	actin related protein 1 [*Pinus massoniana*]	12.518	6	6
WCL24807.1	photosystem II 44 kDa protein (chloroplast) [*Pinus massoniana*]	8.537	6	6
WCL24370.1	photosytem I subunit VII (chloroplast)	8.045	5	5
WCL24671.1	photosystem II protein D1 (chloroplast) [*Pinus massoniana*]	6.064	4	4
WCL25227.1	photosystem II 47 kDa protein (chloroplast) [*Pinus massoniana*]	5.584	4	4
AIZ74332.1	beta-tubulin [*Pinus massoniana*]	4.894	4	4
AIZ74331.1	alpha-tubulin [*Pinus massoniana*]	6.451	3	3
WCL23846.1	photosystem II protein D2 (chloroplast) [*Pinus massoniana*]	4.799	3	3
ACY66805.1	chlorophyll a/b-binding protein [*Pinus massoniana*]	4.253	3	3
AHJ86267.1	glutathione peroxidase [*Pinus massoniana]*	5.426	14	2
AIZ74335.1	polyubiquitin 3, partial [*Pinus massoniana*]	2.894	2	2
WCL24189.1	ATP synthase CF1 epsilon subunit (chloroplast) [*Pinus massoniana*]	2.562	2	2
WCL23911.1	ribosomal protein L2 (chloroplast) [*Pinus massoniana*]	4.188	1	1
AIZ74341.1	isocitrate dehydrogenase [*Pinus massoniana*]	3.581	1	1
WCL38145.1	photosystem I P700 chlorophyll a apoprotein A1 (chloroplast) [*Pinus massoniana*]	2.016	1	1
ACV88654.1	cyclophilin [*Pinus massoniana*]	1.319	1	1
WCL24138.1	photosystem I P700 chlorophyll a apoprotein A2 (chloroplast) [*Pinus massoniana*]	1.117	1	1
WCL25009.1	cytochrome b6 (chloroplast) [*Pinus massoniana*]	1.06	1	1
WCL25872.1	ATP-dependent Clp protease proteolytic subunit (chloroplast) [*Pinus massoniana*]	0.863	1	1
AMR43653.1	purple acid phosphatase 1 [*Pinus massoniana*]	0.861	1	1
WCL25530.1	ribosomal protein S11 (chloroplast) [*Pinus massoniana*]	0.861	1	1
AIF75959.1	putative phosphofructokinase, partial [*Pinus massoniana*]	0.842	1	1
AVP71779.1	auxin response factor 16 [*Pinus massoniana*]	0.763	3	1
UFA45708.1	bHLH10 [*Pinus massoniana*]	0.684	1	1
WCL23782.1	Ycf2 (chloroplast) [*Pinus massoniana*]	0.677	1	1
AHL67654.1	caffeoyl-CoA 3-O-methyltransferase [*Pinus massoniana*]	0.635	5	1
AIF75747.1	dehydrin 1 protein, partial [*Pinus massoniana*]	0.621	5	1
WCL25253.1	hypothetical chloroplast RF68 (chloroplast) [*Pinus massoniana*]	0.595	1	1
UIB01906.1	2-C-methyl-D-erythritol 4-phosphate cytidylyltransferase [*Pinus massoniana*]	0.584	1	1

## Data Availability

All data generated during this study are included in this published article, and the raw data used or analyzed during the current study are available from the corresponding author on reasonable request.

## Supplementary Materials

The following supporting information can be downloaded at: https://www.mdpi.com/article/10.3390/plants13192672/s1, Table S1: The binding proteins of the *PmACRE1* gene promoter in *Pinus massoniana*; Figure S1: Regulatory Cis-elements identify in the *PmACRE1* gene Promoter Sequence; Figure S2: Natural soluble proteins extracted from the stem and needle leaves of *Pinus massoniana* inoculated with pine wood nematode; Figure S3: Natural soluble proteins extracted from the needle leaves of *Pinus massoniana* inoculated with pine wood nematode.
