# Supplementary material for: Transcription Factor and Protein Regulatory Network of PmACRE1 in Pinus massoniana Response to Pine Wilt Nematode Infection
Source: Plants (Basel). 2024 Sep 24;13(19):2672. doi: 10.3390/plants13192672 (PMC11479228; doi:10.3390/plants13192672)
Supplement: Supplementary file 1 [file plants-13-02672-s001.zip › Supplementary Table S1.pdf]

Supplementary Table

Supplementary Table S1 The binding proteins of the *PmACRE1* gene promoter in *Pinus massoniana*

Accession	Description	Sum PEP Score	Peptides	Unique Peptides
CL27073.1	ribulose-1,5-bisphosphate carboxylase/oxygenase large subunit (chloroplast) [*Pinus tabuliformis var. henryi*]	63.807	18	6
AXQ01702.1	ATP synthase CF1 alpha subunit (plastid) [*Pinus thunbergii*]	54.983	17	15
YP_009388265.1	ATP synthase CF1 beta chain (chloroplast) [*Pinus sylvestris*]	81.316	16	6
BAD32766.1	ribulose-1,5-bisphosphate carboxylase/oxygenase large subunit, partial (chloroplast) [*Pinus clausa*]	70.795	16	2
AET45456.1	cytochrome f (chloroplast) [*Pinus taiwanensis*]	65.158	15	15
AAA33780.1	glyceraldehyde-phosphate dehydrogenase [*Pinus sylvestris*]	42.237	15	8
AQX43169.1	Ferredoxin-dependent glutamate synthase [*Pinus pinaster*]	28.036	14	9
AXY96135.1	ATPase subunit 1 (mitochondrion) [*Pinus taeda*]	26.446	13	11
AAA33779.1	glyceraldehyde-3-phosphate dehydrogenase [*Pinus sylvestris*]	32.044	12	9
ABA02402.1	ribulose-1,5-bisphosphate carboxylase/oxygenase large subunit, partial (chloroplast) [*Tsuga mertensiana*]	48.972	11	2
ULQ63855.1	ribulose-1,5-bisphosphate carboxylase/oxygenase large subunit (chloroplast) [*Cuscuta japonica*]	47.31	11	1
UBR19730.1	cobalamin-independent methionine synthase 3 [*Pinus pinaster*]	25.511	11	7
CCJ32336.1	ribulose-1,5-bisphosphate carboxylase/oxygenase large subunit, partial [*Pinus nigra subsp. nigra*]	43.455	10	1
AFA51418.1	extracellular calcium sensing receptor [*Pinus massoniana*]	28.98	10	10
WCL32873.1	photosystem II 44 kDa protein (chloroplast) [*Pinus tabuliformis*]	40.823	9	4
CAA78900.1	2104448A Lhcb5 gene	24.35	9	9
P10053.1	RecName: Full=Ribulose bisphosphate carboxylase small subunit, chloroplastic; Short=RuBisCO small	47.909	8	4
AAL29462.1	Cu-Zn-superoxide dismutase precursor [*Pinus pinaster*]	38.17	8	7
AZA14769.1	Fasciclin-like arabinogalactan protein 8 [*Pinus taeda*]	29.44	8	7
AAD10215.1	glyceraldehyde-3-phosphate dehydrogenase (chloroplast) [*Pinus sylvestris*]	28.481	8	6

WCL24087.1	ATP synthase CF0 B subunit (chloroplast) [*Pinus massoniana*]	19.527	8	8
BAD72793.1	glyceraldehyde-3-phosphate dehydrogenase, partial [*Pinus thunbergii*]	17.63	8	1
CBB98614.1	unnamed protein product [*Pinus pinaster*]	30.74	7	5
AUR27414.1	ribulose-1,5-bisphosphate carboxylase/oxygenase large subunit, partial (chloroplast) [*Festuca brachyphylla*]	29.139	7	2
ADX94851.1	photosystem II protein D1 (chloroplast) [*Pinus monophylla*]	25.527	7	7
AZA14770.1	Fasciclin-like arabinogalactan protein 8 [*Pinus taeda*]	25.114	7	6
CBL95261.1	malate dehydrogenase [*Pinus pinaster*]	22.229	7	5
CCC55420.1	mitochondrial serine hydroxymethyltransferase [*Pinus pinaster*]	19.294	7	7
CAW96629.1	unnamed protein product [*Pinus pinaster*]	25.164	6	6
ULQ63839.1	photosystem II CP43 chlorophyll apoprotein (chloroplast) [*Cuscuta japonica*]	23.57	6	1
CAA56286.1	glutamate synthase (ferredoxin), partial [*Pinus sylvestris*]	16.002	6	1
WCL24370.1	photosytem I subunit VII (chloroplast) [*Pinus massoniana*]	15.806	6	6
CAC84682.1	putative histone H1 [*Pinus pinaster*]	15.24	6	5
WCL33387.1	photosystem I P700 chlorophyll a apoprotein A1 (chloroplast) [*Pinus tabuliformis*]	13.662	6	6
ABQ14358.1	putative cyclophilin [*Pinus taeda*]	11.032	6	6
AFG57273.1	hypothetical protein 2_2585_01, partial [*Pinus taeda*]	23.26	5	5
CCC55419.1	mitochondrial glycine decarboxylase complex H-protein [*Pinus pinaster*]	20.239	5	5
AAB19040.1	type 2 light-harvesting chlorophyll a/b-binding polypeptide, partial [*Pinus palustris*]	19.805	5	3
CAA32657.1	unnamed protein product [*Pinus sylvestris*]	17.284	5	2
AAX49666.1	ribulose-1,5-bisphosphate carboxylase/oxygenase large subunit, partial (chloroplast) [*Pinus virginiana*]	17.112	5	1

CCC42221.1	vitamin-b12 independent methionine synthase [*Pinus pinaster*]	16.899	5	1
CBC51097.1	unnamed protein product [*Pinus taeda*]	16.163	5	2
AAR05796.1	NADP+isocitrate dehydrogenase [*Pinus pinaster*]	12.333	5	4
ABR15472.1	UDP-glucose pyrophosphorylase [*Pinus taeda*]	11.399	5	5
CAC84492.1	putative ribulose bisphosphate carboxylase small chain, partial [*Pinus pinaster*]	10.923	5	1
AEX11389.1	hypothetical protein 0_13794_01, partial [*Pinus taeda*]	15.563	5	3
ACI90635.1	chloroplast light harvesting chlorophyll a/b binding protein, partial [*Pinus yunnanensis*]	13.15	5	2
AET45414.1	photosystem I P700 chlorophyll a apoprotein A2 (chloroplast) [*Pinus tropicalis*]	12.648	5	5
AQX42910.1	cytosolic glutamine synthetase GS1a [*Pinus pinaster*]	10.905	5	1
7Y55	A Chain A, PdGSTU1	9.064	5	4
CAA49476.1	glutamate--ammonia ligase [*Pinus sylvestris*]	7.356	5	1
CAA41454.1	CuZn superoxide dismutase [*Pinus sylvestris*]	24.352	4	4
CAC83359.1	histone H2B protein, partial [*Pinus pinaster*]	22.873	4	4
KAG8480668.1	hypothetical protein CXB51_025221 [*Gossypium anomalum*]	21.503	4	1
Q9M612.1	RecName: Full=Nascent polypeptide-associated complex subunit alpha-like protein; Short=NAC-alpha-	20.294	4	1
AFG43734.1	hypothetical protein 2_3006_02, partial [Pinus taeda]	18.75	4	4
ADP03265.1	malate dehydrogenase, partial [Pinus sylvestris]	15.281	4	3
ALK01132.1	photosystem II protein D2 (chloroplast) [*Gnetum gnemon*]	15.118	4	1
AET48001.1	photosystem II protein D2 (chloroplast) [*Pinus johannis*]	13.494	4	1
ATP73033.1	hypothetical protein 2_4107_01, partial [*Pinus sylvestris*]	13.122	4	4

ADQ37306.1	putative flavoprotein-containing polyamine oxidase, partial [*Pinus sylvestris*]	12.344	4	4
CAC84681.1	putative histone H2B [*Pinus pinaster*]	12.29	4	1
AAU89255.1	chloroplast light harvesting chlorophyll a/b binding protein, partial [*Pinus roxburghii*]	11.571	4	2
CCC55430.1	cytosolic serine hydroxymethyltransferase [*Pinus pinaster*]	10.714	4	4
ATP74727.1	hypothetical protein UMN_CL132Contig1_03, partial [*Pinus sylvestris*]	10.564	4	3
CAC84493.1	putative nucleoside diphosphate kinase [*Pinus pinaster*]	9.161	4	4
ACB11496.1	catalase [*Pinus sylvestris*]	6.746	4	4
CAC83306.1	putative NAD-dependent formate dehydrogenase, partial [*Pinus pinaster*]	4.264	4	4
CAA41406.1	Type II chlorophyll a /b-binding protein [*Pinus sylvestris*]	22.02	3	1
KAG8490228.1	hypothetical protein CXB51_016059 [*Gossypium anomalum*]	18.519	3	1
CAA41407.1	Type III chlorophyll a /b-binding protein [Pinus sylvestris]	14.499	3	2
AFG61479.1	hypothetical protein CL1154Contig1_04, partial [*Pinus taeda*]	12.314	3	2
AAG45937.1	ascorbate peroxidase, partial [*Pinus strobus*]	11.456	3	1
AAL74386.1	LHC I type II chlorophyll binding protein, partial [*Pinus sylvestris*]	11.391	3	1
CAW38451.1	unnamed protein product [*Pinus pinaster*]	11.308	3	1
AIZ74335.1	polyubiquitin 3, partial [*Pinus massoniana*]	11.156	3	3
AFG63675.1	hypothetical protein 0_18315_01 [*Pinus taeda*]	10.953	3	3
CAA78932.1	Lhca4 protein,Type 4 protein of light-harvesting complex of photosystem I [*Pinus sylvestris*]	9.584	3	3
AFG45778.1	hypothetical protein CL265Contig2_04, partial [*Pinus taeda*]	9.409	3	3
AGT98545.1	glutathione peroxidase 4 [*Pinus tabuliformis*]	8.492	3	2

AFG69966.1	hypothetical protein CL1136Contig1_04, partial [*Pinus taeda*]	8.276	3	3
AFG66318.1	hypothetical protein 0_14402_01, partial [*Pinus taeda*]	8.081	3	3
ALK01037.1	cytochrome b6 (chloroplast) [*Abies sibirica*]	7.564	3	3
AAG17036.1	S-adenosylmethionine synthetase [*Pinus contorta*]	6.92	3	3
AAC60560.2	NADPH-protochlorophyllide-oxidoreductase [*Pinus mugo*]	6.749	3	3
QDZ06018.1	putative triosephosphate isomerase, partial [*Pinus contorta var. latifolia*]	6.603	3	3
CAW60977.1	unnamed protein product [*Pinus taeda*]	6.389	3	1
QWM97365.1	non-specific lipid transfer protein [*Pinus sylvestris*]	6.122	3	3
AAG01147.1	calreticulin [*Pinus taeda*]	6.086	3	3
AAD09590.1	calmodulin protein, partial [*Pinus taeda*]	5.006	3	2
AET45106.1	ATP synthase, F0 complex, c subunit (chloroplast) [*Pinus yecorensis*]	4.982	3	3
ACJ70303.1	putative phytocyanin [*Pinus sylvestris*]	4.223	3	3
AEW08137.1	hypothetical protein 2_784_02, partial [*Pinus radiata*]	12.503	2	1
P81664.1	RecName: Full=Phosphoribulokinase; Short=PRK; Short=PRKase; AltName: Full=Phosphopentokinase	11.769	2	2
P84722.1	RecName: Full=Putative fructose-bisphosphate aldolase, chloroplastic; AltName: Full=PS6	9.312	2	2
AFG67566.1	hypothetical protein CL212Contig1_02, partial [*Pinus taeda*]	7.783	2	1
CAC84677.1	putative histone H2A [*Pinus pinaster*]	7.686	2	1
AEW08533.1	hypothetical protein CL305Contig1_05, partial [*Pinus lambertiana*]	6.332	2	2
CAC84488.1	putative cytokinin-repressed protein [*Pinus pinaster*]	6.329	2	2
AEW08753.1	hypothetical protein CL1530Contig1_04, partial [*Pinus lambertiana*]	6.073	2	2

AFG59652.1	hypothetical protein 2_9400_01, partial [*Pinus taeda*]	6.067	2	2
AFG54417.1	hypothetical protein CL2416Contig1_03, partial [*Pinus taeda*]	5.937	2	1
AEW09147.1	hypothetical protein CL4276Contig1_01, partial [*Pinus radiata*]	5.935	2	2
UTF00587.1	glyceraldehyde-3-phosphate dehydrogenase C, partial [*Pinus sylvestris*]	5.627	2	1
AFG52515.1	hypothetical protein 2_8083_01, partial [*Pinus taeda*]	5.617	2	2
AFG66353.1	hypothetical protein 0_2313_02, partial [*Pinus taeda*]	5.328	2	2
YP_009388195.1	cytochrome b559 alpha chain (chloroplast) [*Juniperus communis*]	4.929	2	1
AFG44729.1	hypothetical protein CL106Contig1_04, partial [*Pinus taeda*]	4.868	2	1
AHY28824.1	chloroplast Cu/Zn superoxide dismutase, partial [*Pinus echinata*]	4.391	2	1
AGT98544.1	glutathione peroxidase 3 [*Pinus tabuliformis*]	4.369	2	1
AFG70655.1	hypothetical protein 2_945_01, partial [*Pinus taeda*]	4.366	2	1
AAW79090.1	calcium-dependent protein kinase, partial [*Pinus taeda*]	4.196	2	2
AEW09110.1	hypothetical protein CL3992Contig1_03, partial [*Pinus radiata*]	4.16	2	2
ADV18608.1	EF-hands-containing protein, partial [*Pinus mugo*]	4.047	2	2
AEW08720.1	hypothetical protein CL1360Contig1_05, partial [*Pinus lambertiana*]	4.034	2	1
AXR86084.1	PSII cytochrome b559 alpha chain (chloroplast) [*Pinus wangii*]	3.96	2	1
CBD08218.1	unnamed protein product [*Pinus radiata*]	3.873	2	2
ABB02394.1	temperature-induced lipocalin [*Pinus taeda*]	3.531	2	2
ADP03055.1	6-phosphogluconate dehydrogenase [*Pinus sylvestris*]	3.407	2	2
AJP06229.1	AGO5 [*Pinus tabuliformis*]	3.097	2	2

AFG54093.1	hypothetical protein 0_18283_02, partial [*Pinus taeda*]	3.005	2	2
AEW08969.1	hypothetical protein CL2576Contig1_01, partial [*Pinus lambertiana*]	2.752	2	2
AJP06248.1	BSU2 [Pinus tabuliformis]	2.719	2	2
AEW08577.1	hypothetical protein CL593Contig1_05, partial [*Pinus radiata*]	2.67	2	1
AJP06326.1	MCE1 [*Pinus tabuliformis*]	2.622	2	2
UIB01912.1	isopentenyl diphosphate delta-isomerase [*Pinus massoniana*]	2.598	2	2
CBL95265.1	alcohol dehydrogenase [*Pinus pinaster*]	2.53	2	2
AGC13119.1	tau class glutathione S-transferase [*Pinus tabuliformis*]	2.458	2	1
AFG64163.1	hypothetical protein CL4262Contig1_02, partial [*Pinus taeda*]	2.35	2	2
AFG62910.1	hypothetical protein 2_2559_01, partial [*Pinus taeda*]	2.283	2	2
AEW07855.1	hypothetical protein 0_13217_02, partial [*Pinus lambertiana*]	2.043	2	2
ABR15470.1	sucrose synthase [*Pinus taeda*]	1.673	2	2
AFG52023.1	hypothetical protein UMN_3613_01, partial [*Pinus taeda*]	1.326	2	2
CAM12501.1	small heat-shock protein, partial [*Pinus taeda*]	2.683	1	1
AGC13144.1	EF1Bgamma class glutathione S-transferase [*Pinus tabuliformis*]	0.757	1	1
AEX12968.1	hypothetical protein CL192Contig1_03, partial [*Pinus taeda*]	0.755	1	1
AEX11200.1	hypothetical protein 0_12402_01, partial [*Pinus taeda*]	0.654	1	1
ABB78088.1	1-hydroxy-2-methyl-2-(E)-butenyl 4-diphosphate reductase type 1 [*Ginkgo biloba*]	0.593	1	1
