# Supplementary figures and images for: Transcription Factor and Protein Regulatory Network of PmACRE1 in Pinus massoniana Response to Pine Wilt Nematode Infection
Source: Plants (Basel). 2024 Sep 24;13(19):2672. doi: 10.3390/plants13192672 (PMC11479228; doi:10.3390/plants13192672)

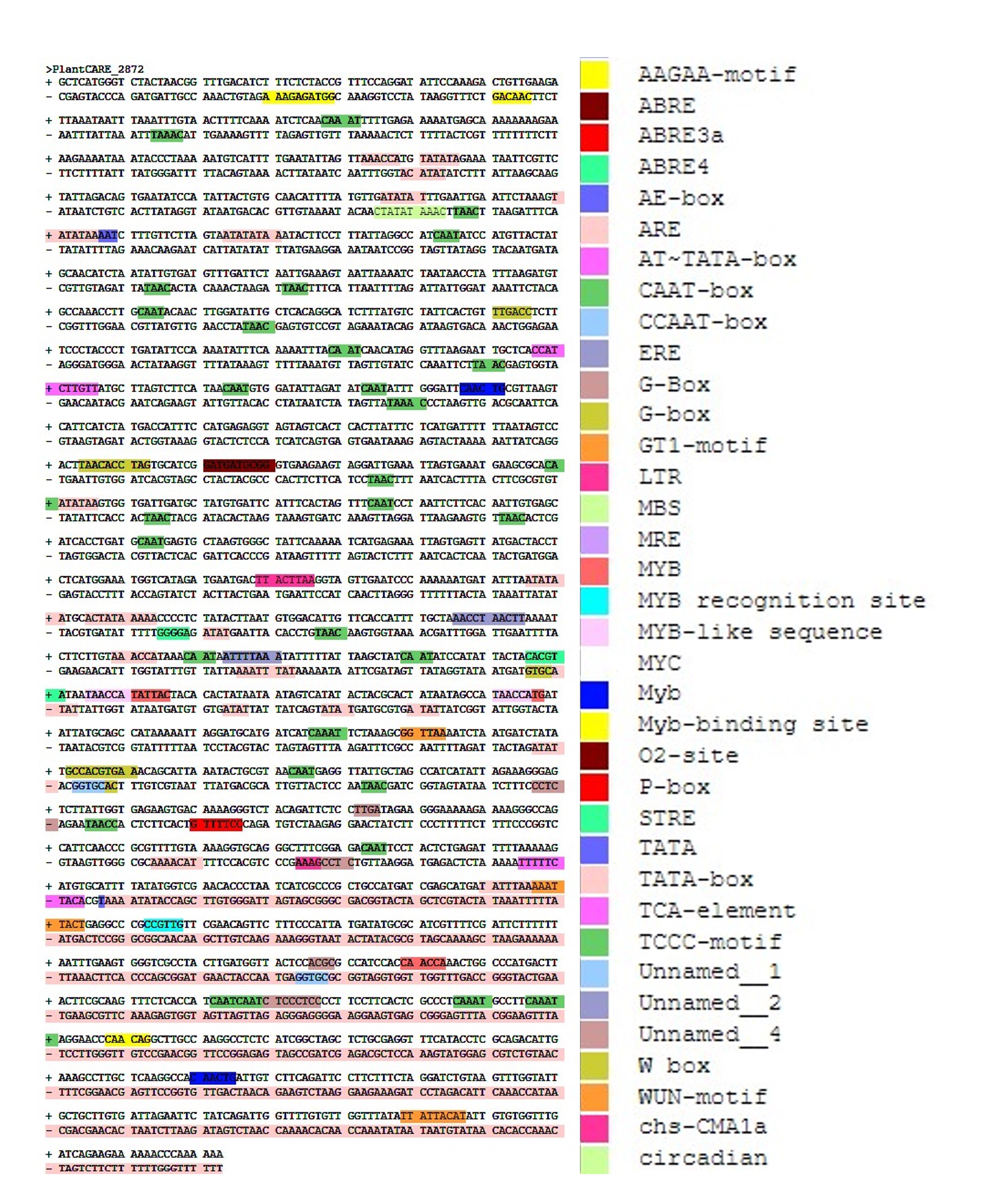

Supplement: Supplementary file 1 [file plants-13-02672-s001.zip › Supplementary Figure S1.jpg]

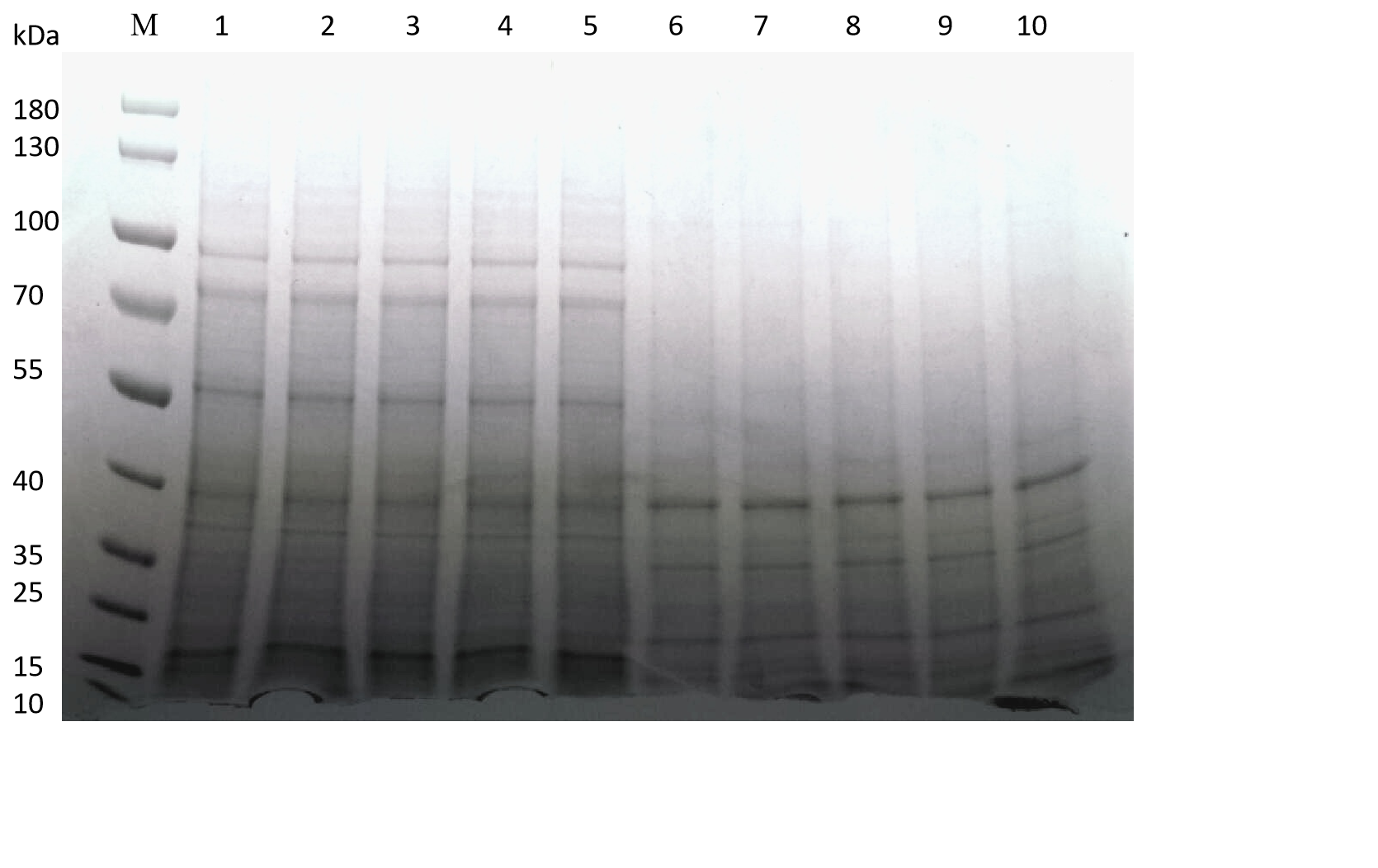

Supplement: Supplementary file 1 [file plants-13-02672-s001.zip › Supplementary Figure S2.tif]

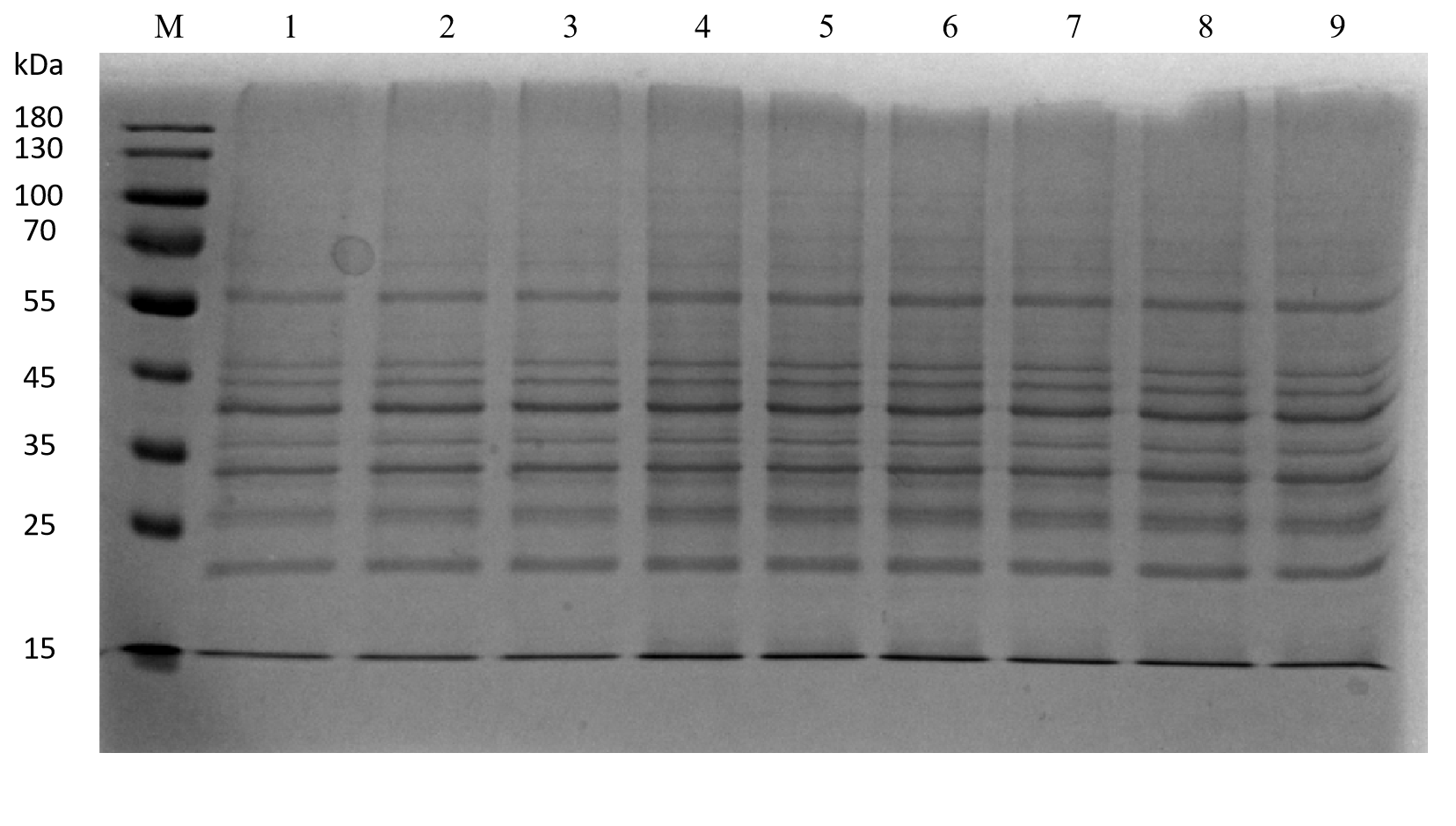

Supplement: Supplementary file 1 [file plants-13-02672-s001.zip › Supplementary Figure S3.tif]
